# DEVELOPMENT AND VALIDATION OF A MORTALITY PREDICTION MODEL AMONG MIDDLE-AGED AND OLDER FRAIL ADULTS: UK BIOBANK

**DOI:** 10.1093/geroni/igae098.1773

**Published:** 2024-12-31

**Authors:** Chenkai Wu, Jianhong Xu, Yanxin Wang

**Affiliations:** Duke Kunshan University, Kunshan, Jiangsu, China (People’s Republic); Duke Kunshan University, Kunshan, Jiangsu, China (People’s Republic); Duke Kunshan University, Kunshan, Jiangsu, China (People’s Republic)

## Abstract

Research has identified numerous risk factors for frailty and developed prediction models for the onset of frailty. However, little is known about how multimodal risk factors collectively influence the prognosis of frailty. We developed and validated prediction models for mortality risk among the frail during a 10-year period. Data were from 15,404 frail individuals aged 37-73 years from the UK Biobank. The entire sample was divided into development set (n=14,272 from England and Wales) and validation set (n=1,132 from Scotland). We assessed the associations of 383 survey, 144 physical, and 59 biomarker predictors with all-cause mortality. Variable selection was conducted using LASSO and stepwise regression. We built a series of nested models: survey only (Model 1), survey+physical measurement (Model 2), and survey+physical measurement+biomarker (Model 3). Model performance was measured by discrimination, calibration, and reclassification. A total of 5,654 males and 9,750 females (63.3%) were included. Model 1 retained 122 survey predictors, Model 2 added 42 physical measurements, and Model 3 added 60 biomarkers. The C-statistic was 0.77, 0.80, and 0.79 in three models, respectively. Models 2 and 3 had improvement in reclassification in mortality risk over Model 1 (net reclassification index=0.111 and 0.029). The light gradient-boosting machine found age, cystatin C, FEV1, smoking, and employment status to be most important predictors. We have developed and validated a series of prediction models using multi-modal data to quantify mortality risk among the frail. The findings provide valuable insights into the design of patient-targeted intervention and management strategies.

